# *Notes from the Field*: Potential Outbreak of Extrapulmonary *Mycobacterium abscessus* subspecies *massiliense* Infections from Stem Cell Treatment Clinics in Mexico — Arizona and Colorado, 2022

**DOI:** 10.15585/mmwr.mm7318a3

**Published:** 2024-05-09

**Authors:** Minh-Vu H. Nguyen, Nabeeh A. Hasan, Vinicius Calado Nogueira De Moura, L. Elaine Epperson, Christopher A. Czaja, Helen Johnston, Nicholas Laramee, Kelsey Orten, JulieAnna Rivas, Siru Prasai, Marissa K. Grossman, Kiran M. Perkins, David E. Griffith, Reeti Khare, Michael Strong, Charles L. Daley

**Affiliations:** ^1^Division of Mycobacterial and Respiratory Infections, Department of Medicine, National Jewish Health, Denver, Colorado; ^2^Center for Genes, Environment and Health, National Jewish Health, Denver, Colorado; ^3^Colorado Department of Public Health and Environment; ^4^Arizona Department of Health Services; ^5^Maricopa County Department of Public Health, Phoenix, Arizona; ^6^Division of Healthcare Quality Promotion, National Center for Emerging and Zoonotic Infectious Diseases, CDC; ^7^Epidemic Intelligence Service, CDC; ^8^Advanced Diagnostics Laboratories, National Jewish Health, Denver, Colorado; ^9^Division of Infectious Diseases, Department of Medicine, University of Colorado, Aurora, Colorado; ^10^Division of Pulmonary Sciences and Critical Care, Department of Medicine, University of Colorado, Aurora, Colorado.

**Summary**

**What is already known about this topic?**

*Mycobacterium abscessus* is a difficult-to-treat nontuberculous mycobacterium; various extrapulmonary infections have been reported associated with medical tourism.

**What is added by this report?**

In 2022, three patients developed extrapulmonary *M. abscessus* infections after receiving embryonic stem cell injections in three cities in Mexico. Isolates from two patients were identified as *M. abscessus* subspecies *massiliense* of a single clone, distinct from known dominant circulating clones (DCCs), by whole genome sequencing.

**What are the implications for public health practice?**

Because these isolates were clonal, distinct from known DCCs, and derived from distant cities, a common infected source associated with embryonic stem cell injections is suspected. Vigilance for similar cases and guidance for persons considering medical tourism are advised.

*Mycobacterium abscessus* is an intrinsically drug-resistant, rapidly growing, nontuberculous mycobacterium; extrapulmonary infections have been reported in association with medical tourism (*1*). During November–December 2022, two Colorado hospitals (hospitals A and B) treated patient A, a Colorado woman aged 30–39 years, for *M. abscessus* meningitis. In October 2022, she had received intrathecal donor embryonic stem cell injections in Baja California, Mexico to treat multiple sclerosis and subsequently experienced headaches and fevers, consistent with meningitis. Her cerebrospinal fluid revealed neutrophilic pleocytosis and grew *M. abscessus* in culture at hospital A. Hospital A’s physicians consulted hospital B’s infectious diseases (ID) physicians to co-manage this patient (*2*).

In spring 2023, hospital B’s ID physicians identified two additional patients with *M. abscessus* infections acquired after receiving stem cell injections performed at different clinics in Mexico. The first of these, patient B, an Arizona man aged 60–69 years, developed a right elbow osteoarticular infection after receiving donor embryonic stem cell injections for psoriatic arthritis at a Baja California, Mexico clinic different from the one that treated patient A, in April 2022. The second, patient C, a Colorado man aged 60–69 years, developed bilateral knee infections after receiving donor embryonic stem cell injections in both knees for osteoarthritis at a clinic in Guadalajara, Mexico, in October 2022.

## Investigation and Outcomes

Hospital B’s ID physicians requested the isolates but were only able to obtain the original isolates from patients A and B. Whole genome sequencing (WGS) and phylogenetic analysis, performed at hospital B (*3*), revealed that the isolates were clonal *M. abscessus* subspecies *massiliense*, with only one single nucleotide polymorphism (SNP) difference between the two isolate core genomes* and were distinct from the most prevalent characterized dominant circulating clones (*3*) (Figure). These isolates were regrown from their original subcultures to repeat WGS, and the one SNP difference was confirmed. These patients had their stem cell injections performed at clinics 167 miles (269 km) apart in Baja California, Mexico. As of March 28, 2024, treatment is ongoing for all three patients.

**FIGURE:**
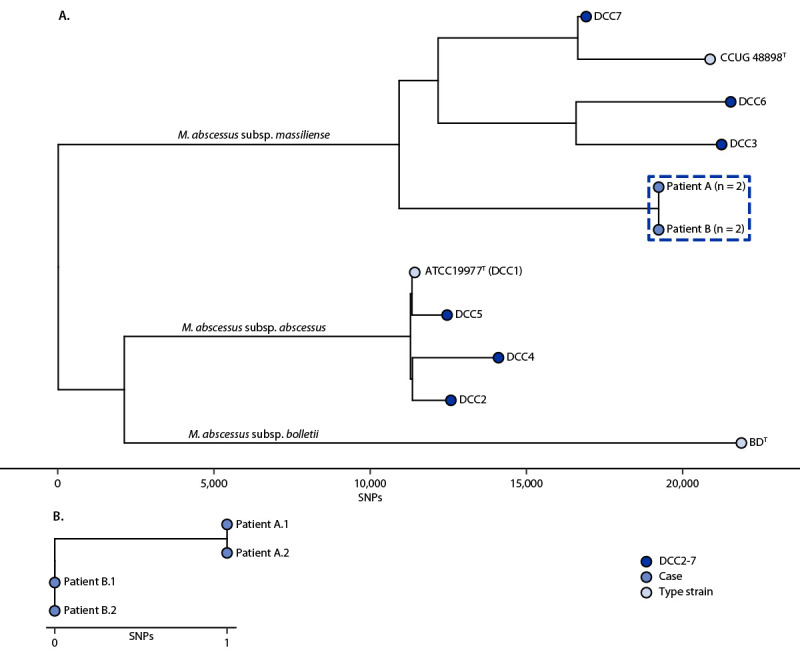
*Mycobacterium abscessus* whole genome phylogeny*^,^^†^ of dominant circulating clones 1–7 and isolates from patients A^§^ and B^¶^ (A) and genomic similarity between the first and second whole genome sequencing single nucleotide polymorphisms for isolates from patients A^§^ and B^¶^ (B) associated with receipt of stem cell treatment in Mexico — Arizona and Colorado, 2022 **Abbreviations:** ATCC = American type culture collection; BD = Becton Dickinson; DCC = dominant circulating clone; SNP = single nucleotide polymorphism; WGS = whole genome sequencing. * WGS was conducted twice to confirm the SNP difference between isolates from patients A and B. **^†^** Superscripted T (^T^) is the standard designation for a type strain of a taxon. **^§^** Patient A.1 = isolate from patient A, first WGS result; patient A.2 = isolate from patient A, second WGS result. **^¶^** Patient B.1 = isolate from patient B, first WGS result; patient B.2 = isolate from patient B, second WGS result.

The patients’ treating physicians informed their state public health departments of their findings. The Colorado Department of Public Health and Environment (CDPHE) interviewed patients A and C, and the Arizona Department of Public Health interviewed patient B. CDPHE searched for similar cases within Colorado and other states and consulted with CDC; as of March 28, 2024, no additional cases have been identified. This activity was reviewed by CDC, deemed not research, and was conducted consistent with applicable federal law and CDC policy.^†^

## Preliminary Conclusions and Actions

Given that the isolates identified from patients treated at different, distant clinics represent a single clone, the physicians and CDPHE suspect a common infected source (potentially the product, reagents, or equipment used) for patients A and B. CDPHE’s attempts to identify the product or gather details about its administration have been unsuccessful to date. CDPHE attempted to contact clinics that performed the stem cell injections, but received no response. A collaborative process with Mexican health authorities was initiated and is ongoing; however, no new substantial cases have yet been identified.

Next steps include 1) performing WGS on the organism isolated from patient C from newly acquired specimens; 2) sharing genomic information from the WGS analysis with the National Center for Biotechnology Information to ensure that comparisons can be made with additional cases; and 3) conducting prospective case finding. Historically, stem cell treatments have been linked to bacterial infections (*4*), and procedure-related infection risks associated with medical tourism are known (*1*,*5*). Providers and public health agencies need to be aware of the risk for *M. abscessus* infections from stem cell treatments for indications not approved by the Food and Drug Administration and maintain vigilance for similar cases (*5*). They also are advised to provide guidance for persons considering medical tourism (*5*).

## Footnotes

* WGS for isolates from patients A and B have been deposited in the National Center for Biotechnology Information BioProject, CDC HAI-Seq Nontuberculous mycobacterium (542112).

† 45 C.F.R. part 46.102(l)(2), 21 C.F.R. part 56; 42 U.S.C. Sect. 241(d); 5 U.S.C. Sect. 552a; 44 U.S.C. Sect. 3501 et seq.
